# Author Sequence and Credit for Contributions in Multiauthored Publications

**DOI:** 10.1371/journal.pbio.0050018

**Published:** 2007-01-16

**Authors:** Teja Tscharntke, Michael E Hochberg, Tatyana A Rand, Vincent H Resh, Jochen Krauss

## Abstract

A transparent, simple, and straightforward approach that is free from any arbitrary rank valuation is required to estimate the credit associated with the sequence of authors' names on multiauthored papers.

The increasing tendency across scientific disciplines to write multiauthored papers [1,2] makes the issue of the sequence of contributors' names a major topic both in terms of reflecting actual contributions and in a posteriori assessments by evaluation committees. Traditionally, the first author contributes most and also receives most of the credit, whereas the position of subsequent authors is usually decided by contribution, alphabetical order, or reverse seniority. Ranking the first or second author in a two-author paper is straightforward, but the meaning of position becomes increasingly arbitrary as the number of authors increases beyond two. Criteria for authorship have been discussed at length, because of the inflationary increase in the number of authors on papers submitted to biomedical journals and the practice of “gift” authorship [3,4], but a simple way to determine credit associated with the sequence of authors' names is still missing [4–7] (http://www.councilscienceeditors.org).

The situation in our area of research—the ecological and environmental sciences—has changed in recent years. Following informal practices in the biomedical sciences, the last author often gets as much credit as the first author, because he or she is assumed to be the driving force, both intellectually and financially, behind the research. Evaluation committees and funding bodies often take last authorship as a sign of successful group leadership and make this a criterion in hiring, granting, and promotion. This practice is unofficial, and hence not always followed, meaning that sometimes last authors “mistakenly” benefit when they actually are not principal investigators. Moreover, there is no accepted yardstick in assessing the actual contribution of a group leader to given scientific publications [8,9], so interpretation of author sequence can be like a lottery. Hence, one really does not know if being last author means that the overall contribution was the most or least important.

Although reducing evaluation of authors' complex contributions to simple metrics is regrettable, in reality it is already in practice in most evaluation committees. Hence, in our opinion, we need a simple and straightforward approach to estimate the credit associated with the sequence of authors' names that is free from any arbitrary rank valuation. In multiauthored papers, the first author position should clearly be assigned to the individual making the greatest contribution [4–6], as is common practice. However, authors often adopt different methods of crediting contributions for the following authors, because of very different traditions across countries and research fields, resulting in very different criteria that committees adopt to quantify author's contributions [8,9]. For example, some authors use alphabetical sequence, while others think that the last author position has great importance or that the second author position is the second most important. Still others detail each author's contribution in a footnote.

We suggest that the approach taken should be stated in the acknowledgements section, and evaluation committees are asked to weigh the contribution of each author based on the criteria given by the authors. This would make reviewers aware that there are different cultures to authorship order. The usual and informal practice of giving the whole credit (impact factor) to each author of a multiauthored paper is not adequate and overemphasises the minor contributions of many authors (Table 1). Similarly, evaluation of authors according to citation frequencies means often overrating resulting from high-impact but multiauthored publications. The following approaches may be identified.

**Table 1:**
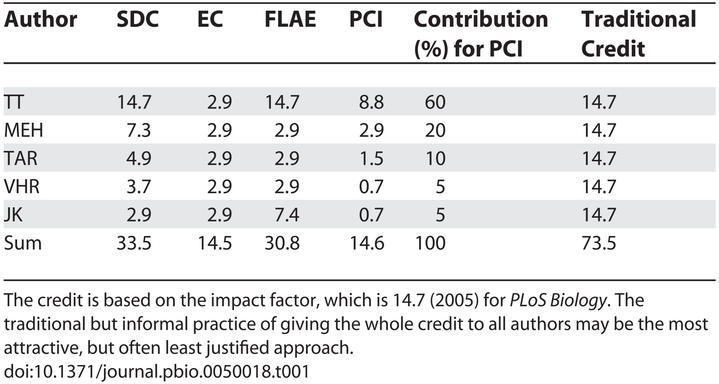
Comparison of the Credit for Contributions to This Paper under the Four Different Models Suggested in the Text

(1) The “sequence-determines-credit” approach (SDC). The sequence of authors should reflect the declining importance of their contribution, as suggested by previous authors [4–6]. Authorship order only reflects relative contribution, whereas evaluation committees often need quantitative measures. We suggest that the first author should get credit for the whole impact (impact factor), the second author half, the third a third, and so forth, up to rank ten. When papers have more than ten authors, the contribution of each author from the tenth position onwards is then valuated just 5%.

(2) The “equal contribution” norm (EC). Authors use alphabetical sequence to acknowledge similar contributions or to avoid disharmony in collaborating groups. We suggest that the contribution of each author is valuated as an equal proportion (impact divided by the number of all authors, but a minimum of 5%).

(3) The “first-last-author-emphasis” norm (FLAE). In many labs, the great importance of last authorship is well established. We suggest that the first author should get credit of the whole impact, the last author half, and the credit of the other authors is the impact divided by the number of all authors [as in (2)].

(4) The “percent-contribution-indicated” approach (PCI). There is a trend to detail each author's contribution (following requests of several journals) [7]. This should also be used to establish the quantified credit.

The SDC approach (as a new suggestion), the EC norm (alphabetical order), the FLAE norm, and the PCI approach may be combined (e.g., FLAE and SDC), but need to be explicitly mentioned in the acknowledgements.

Our suggestion of explicit indication of the method applied, including the simple method of weighing authors' rank in publications in a quantitative way, will avoid misinterpretations and arbitrary a posteriori designations of author contributions. Multidisciplinary scientific collaboration indeed must be encouraged, but we need to avoid misinterpretations so that current and future scientific communities can evaluate author contributions.
